# Application of binding pancreatogastrostomy in laparoscopic central pancreatectomy

**DOI:** 10.1186/1477-7819-10-223

**Published:** 2012-10-26

**Authors:** Hong Defei, Xin Ying, Cai Xiujun, Peng Shuyou

**Affiliations:** 1 Department of Hepatobiliary Surgery, People’s Hospital of Zhejiang Province, No.158, Shang Tang Road, Hangzhou, 310014, China; 2 Department of General Surgery, Sir Run Run Shaw Hospital, Medical School, Zhejiang University, Institute of Micro-invasive Surgery of Zhejiang University, No.3, Qin Chun Road, Hangzhou, 310016, China

**Keywords:** Pancreatic, Pancreaticogastrostomy

## Abstract

**Background:**

The feasibility of binding pancreaticogastrostomy in laparoscopic central pancreatectomy is not known.

**Methods:**

In October 2011, a female patient with a pancreatic neck mass received laparoscopic central pancreatectomy with binding pancreaticogastrostomy.

**Results:**

The operation was successful. No complications occurred. The operative time was 210 min. Blood loss was 120 ml. On day 11 after the operation, the patient was discharged. The postoperative pathological result showed a 2 × 2 × 2-cm solid pseudopapillary tumor of the pancreas with intrapancreatic infiltration. The surgical margin was negative.

**Conclusions:**

Laparoscopic central pancreatectomy with binding pancreaticogastrostomy might be feasible, facilitating further study in laparoscopic pancreatoduodenectomy.

**Trial registration:**

This study was waived from trial registration because it is a retrospective analysis of medical records.

## Background

Pancreatoduodectomy or pancreatic distal resection is usually performed for cases of benign tumors and low-grade malignancy in the pancreatic neck and body. It may lead to postoperative diabetes. At our institution we successfully carried out central pancreatectomy with binding pancreatogastrostomy [1-3]. Recently, we performed the first case of laparoscopic central pancreatectomy with binding pancreatogastrostomy in the world.

## Methods

### Patient

**Figure 1:**
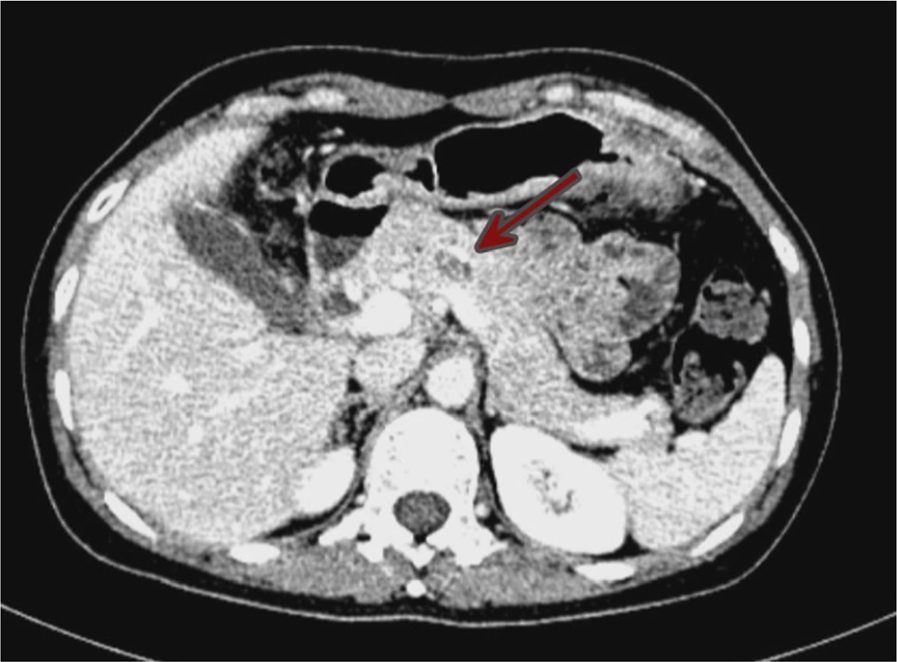
CT demonstrated a 13 × 14-mm, low-density mass in the pancreatic neck.

**Figure 2:**
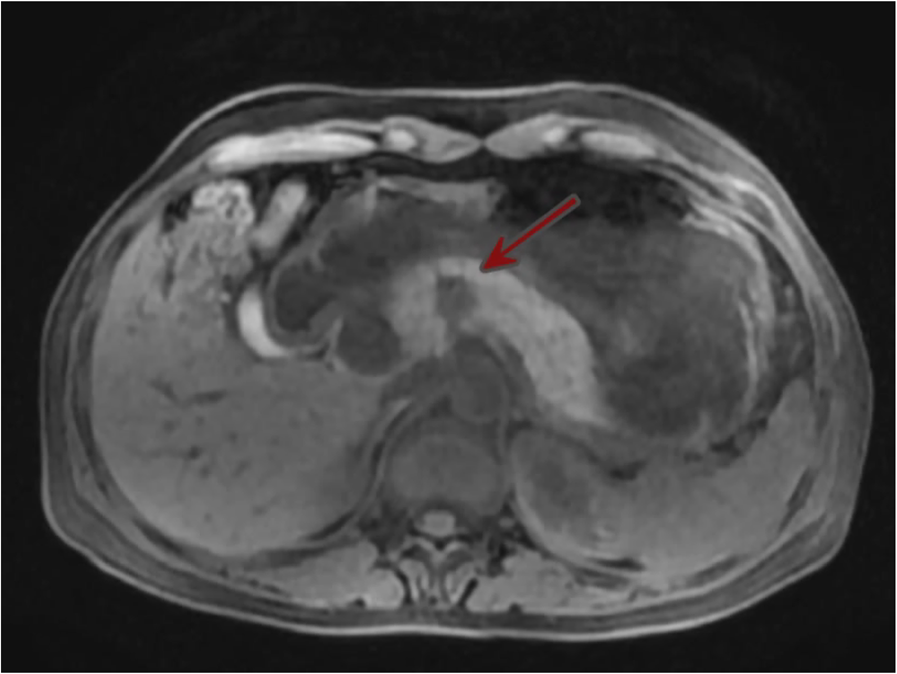
MRI disclosed a mass in the pancreatic neck.

The patient was a 52-year-old female. She was admitted because of epigastric pain for 1 week. Physical examination demonstrated no positive findings. Computed tomography (CT) disclosed a 13 × 14-mm mass in the pancreatic neck with clear margins and mild uneven enhancement (Figure 1). Magnetic resonance imaging (MRI) disclosed a mass in the pancreatic neck (Figure 2). The preoperative diagnosis was a probable cystadenoma or solid pseudopapillary tumor in the pancreatic neck.

### Surgical technique

In October 2011, the patient underwent laparoscopic central pancreatectomy with binding pancreatogastrostomy. The study was approved by the Ethics Committee of Sir Run Run Shaw Hospital of Zhejiang University. The patients signed written informed consent forms concerning the potential surgical risks. The patient was in a supine position and generally anesthetized. A 10-mm incision was made below the umbilicus, and pneumoperitoneum of 15 mmHg was established. After abdominal exploration to rule out metastasis, incisions of 5, 12 and 5 mm were made at the right upper quadrant, left upper quadrant and left lumbar region, respectively.

**Figure 3:**
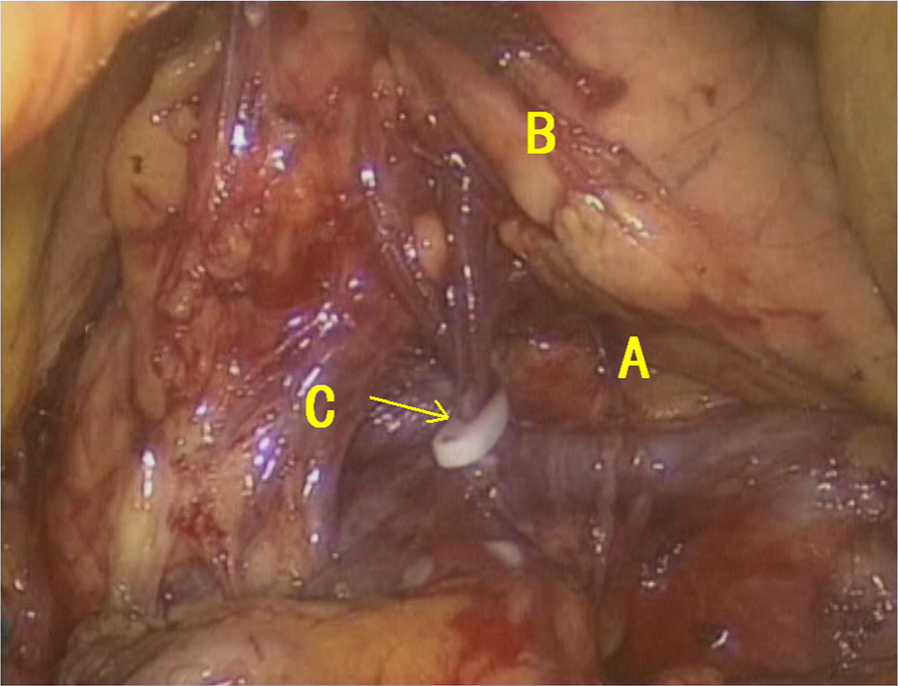
A Splenic vein; B pancreas; C Hemolok.

**Figure 4:**
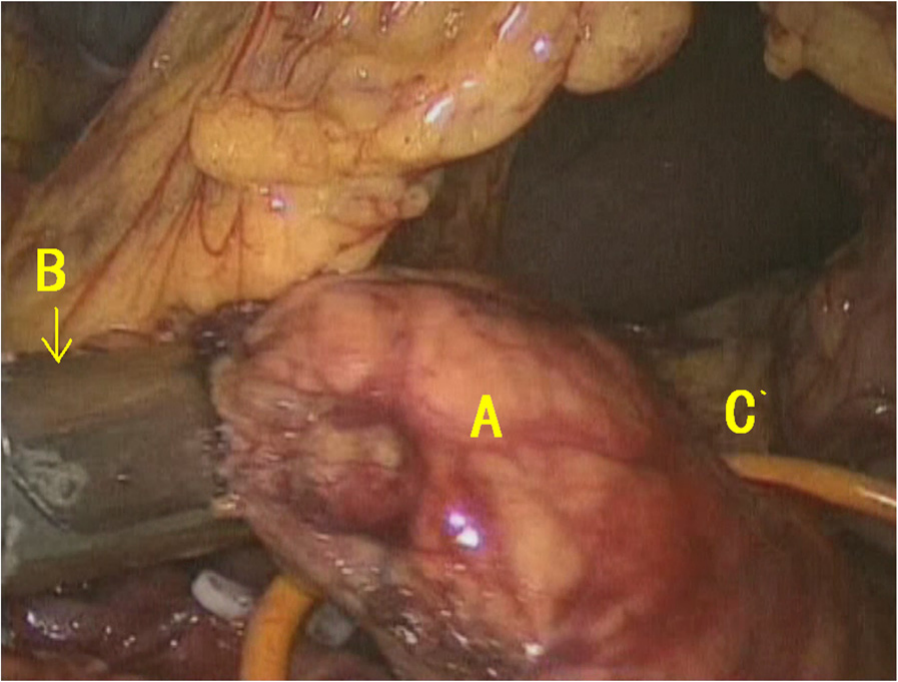
A The central segment of the pancreas; B stapler; C Foley catheter.

First, the gastrocolic ligament was mobilized using a harmonic scalpel. A Foley catheter was applied to suspend the stomach. After the location of the pancreatic tumor had been confirmed, the dissection path below the pancreas was established. The splenic vein and its branches were transected, and the superior mesenteric vein was mobilized thereafter (Figure 3). The retroperitoneum was opened above the pancreas, and the splenic artery was transected. The central segment of the pancreas was lifted upward before staples were used to close the proximal pancreas (Figure 4). A harmonic scalpel was used to transect the distal pancreas with a tumor margin of 2 cm. The frozen section result showed a pancreatic neuroendocrine tumor with negative margins.

**Figure 5:**
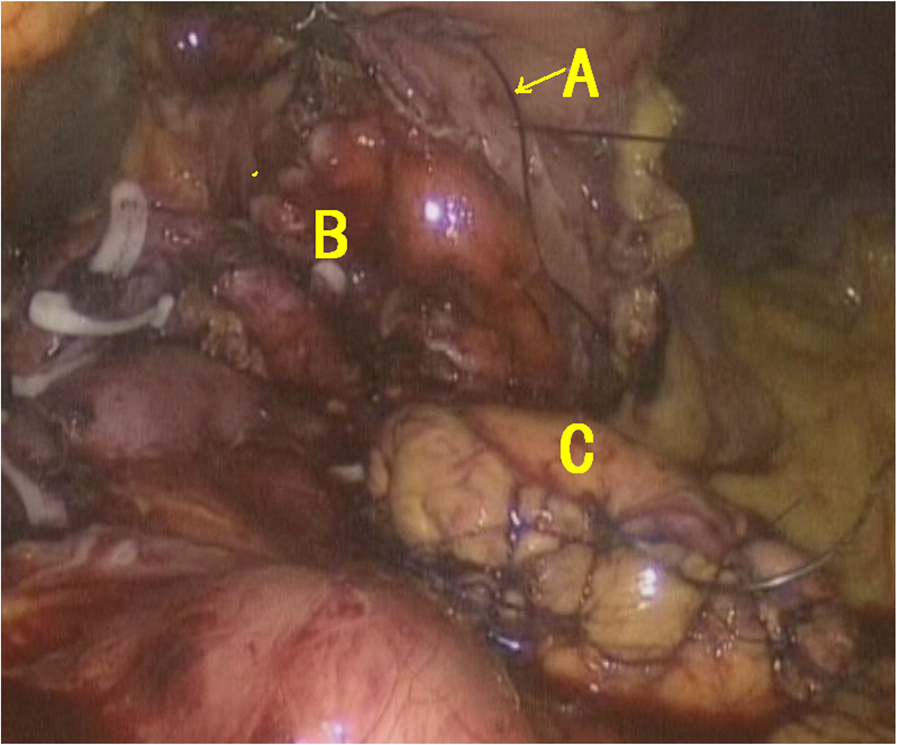
A Purse-string suturing; B incision of the gastric posterior wall; C pancreatic stump.

Secondly, the pancreatic stump was dissected 2 cm distal to the cutting edge; 4–0 prolene sutures were used to stop the bleeding sites on the cutting surface of the pancreas, which also served for retraction at two sides. Povidone-iodine solution was injected into the gastric cavity through a nasogastric tube for sanitization and sucked away thereafter. A patch of the posterior wall of the stomach was removed, and a 4–0 prolene purse-string suture was pre-placed (Figure 5).

**Figure 6:**
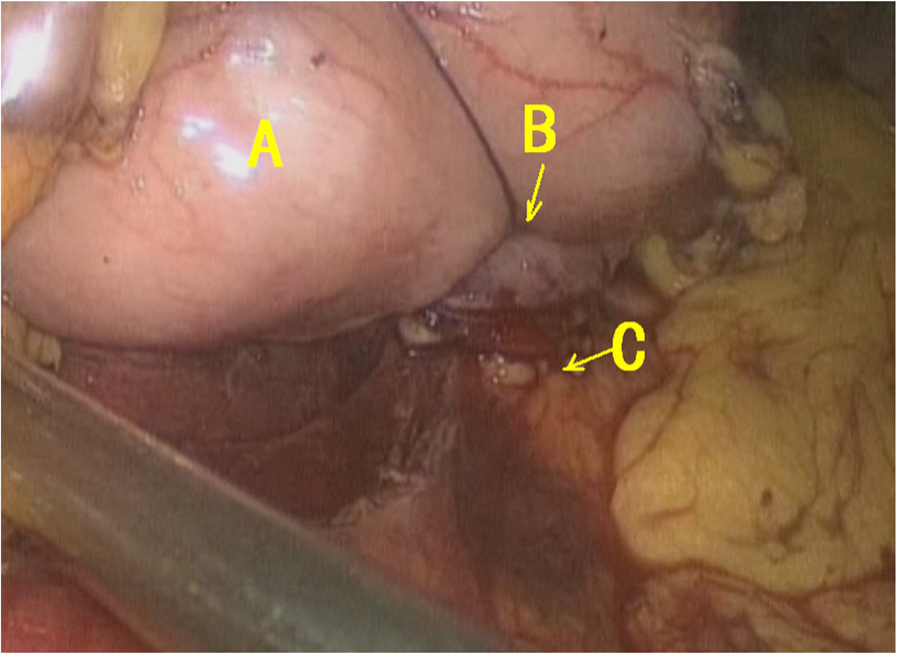
A Gastric posterior wall; B binding purse-string suturing; C pancreatic stump.

**Figure 7:**
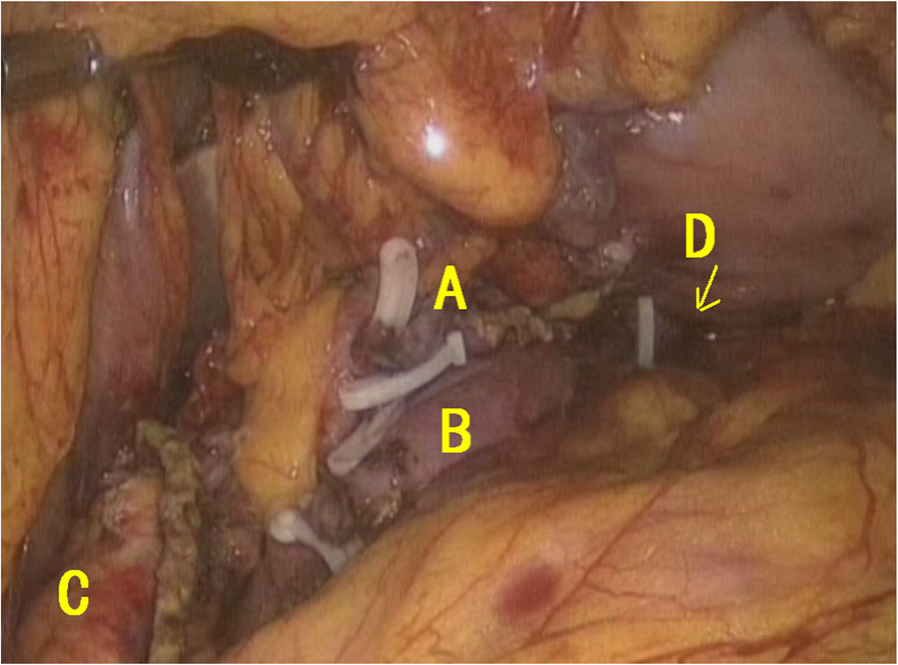
A Splenic artery; B splenic vein; C proximal cutting edge of the pancreas; D pancreatogastrostomy.

The anterior wall of the stomach was opened from where the pancreatic stump was inserted into the gastric cavity. Binding pancreatogastrostomy was completed after the purse-string suture had been fastened (Figure 6). The anterior wall of the stomach was closed, and drainage tubes were placed (Figure 7).

## Results

The operative time was 210 min. The volume of blood loss was 120 ml. Time to return of bowel flatus was 3 days after surgery. The patient started to take in semi-fluid on day 6 after surgery. On day 11 after the operation, the patient was discharged. The postoperative pathological result showed a 2 × 2 × 2-cm solid pseudopapillary tumor of the pancreas with intrapancreatic infiltration. The surgical margin was negative.

## Discussion

Local enucleation may be difficult for benign tumors and low-grade malignancy in the pancreatic neck and body because of large size or being close to the pancreatic duct. For such cases pancreatoduodectomy or pancreatic distal resection was usually performed.

Therefore, the pancreatic parenchyma was sacrificed, which could reach up to 30 to 50% of the total pancreatic tissue in pancreatoduodenectomy [4] and 60 to 90% in pancreatic distal resection [5]. It may lead to postoperative diabetes, with an incidence of 10 to 15% in pancreatoduodenectomy and even higher for cases of chronic pancreatitis with 40%. For pancreatic distal resection the incidence ranged from 25 to 90% in cases with chronic pancreatitis. Postoperative exocrine dysfunction was more common, ranging from 25 to 50% in pancreatoduodenectomy compared to pancreatic distal resection [5].

Central pancreatectomy could preserve the spleen and more pancreatic parenchyma and achieve a better postoperative quality of life. This might be more feasible for cases of benign tumors and low-grade malignancy in the pancreatic neck and body when enucleation is impossible [6].

Because handling of both the proximal and distal pancreatic cutting edges is required, the incidence of postoperative pancreatic leak could reach up to 40%, which is higher than the rate in pancreatectomy and pancreatic distal resection [4]. There are three types of reconstruction for pancreatic stumps: (1) single anastomosis (proximal stump closure plus Roux-en-Y anastomosis of the distal stump and jejunum); (2) double anastomoses (Roux-en-Y anastomosis between both stumps and the jejunum), the so-called “Ω” anastomosis; (3) proximal stump closure plus pancreatogastrostomy between the distal stump and stomach. Few reports in the literature have described side-to-side stump connection [6] or both stumps’ closure without anastomosis [7]. The first procedure was most commonly used. However, pancreatogastrostomy has seldom been studied [6,7].

Non-randomized trials demonstrated a lower incidence of postoperative pancreatic leak and abdominal fluid collection in pancreatogastrostomy compared with pancreatojejunostomy. However, three randomized trials showed no significant difference in pancreatic leak incidence between these two procedures, which was around ten percent [8]. Binding pancreatogastrostomy was originally designed based on the procedures of binding pancreatojejunostomy [9]. We carried out 105 consecutive operations using the open approach, and no leakages occurred [1-3]. This encouraging result urged us to challenge the laparoscopic approach.

Laparoscopic central pancreatectomy with binding pancreatogastrostomy had several advantages, such as being less invasive, preservation of the exocrine and endocrine function of the pancreas, etc. This new procedure was safer since it was not necessary to mobilize splenic vessels in the pancreatic tail [10,11]. The reconstruction was also less time consuming because of the decreased number of anastomoses compared to pancreatoduodenectomy. The pancreatic reconstruction was considered the most challenging part of laparoscopic pancreatoduodenectomy.

## Conclusions

Laparoscopic central pancreatectomy with binding pancreaticogastrostomy might be feasible. Our initial experiences may facilitate further study in laparoscopic pancreatoduodenectomy.

## Abbreviations

CT: Computed tomography; MRI: Magnetic resonance imaging.

## Competing interests

Authors declared there were no competing interests.

## Authors’ contributions

HDF contributed to the concept, study design, data analysis, result interpretation and manuscript writing. XY participated in data collection and the drafting manuscript. CXJ contributed to the study conceptualization and data collection. PSY contributed to conducting the study, data collection and proof reading. All authors read and approved the final manuscript.

## Authors’ information

There was no source of funding for the research and/or publication and no previous communication of this article to a society or meeting.
